# Newborns' Language Discrimination May Not Reflect Sensitivity to Speech Rhythm: Evidence From Computational Modeling

**DOI:** 10.1111/desc.70220

**Published:** 2026-05-08

**Authors:** Ruolan Leslie Famularo, Ali Aboelata, Thomas Schatz, Naomi H. Feldman

**Affiliations:** ^1^ Program in Neuroscience and Cognitive Science University of Maryland College Park Maryland USA; ^2^ Department of Computer Science University of Maryland College Park Maryland USA; ^3^ Department of Linguistics University of Maryland College Park Maryland USA; ^4^ Aix Marseille Univ, CNRS, LIS Marseille France; ^5^ Institute for Advanced Computer Studies University of Maryland College Park Maryland USA

## Abstract

Human newborns are able to discriminate between certain languages but not others. This ability has long been attributed to sensitivity to rhythm—the temporal regularities in speech of different languages. Here, we demonstrate through a series of computational simulations that this discrimination behavior can be achieved using no temporal information at all. Our results raise the possibility that early language discrimination may be independent of speech rhythm, and call for theoretical reconsideration of how infants learn suprasegmental information in the first few months of their lives.

**Summary:**

- We simulate newborn language discrimination with machine learning models on naturalistic speech stimuli.
- Models can discriminate between languages just like newborns, even when rhythmic information is removed.
- This means that infants' behavior could rely on their ability to perceive global properties of speech, rather than rhythm.
- Our results challenge theories of how newborns perceive speech, and how that shapes later language.

## 1 Introduction

Human newborns are able to discriminate between certain languages but not others. This phenomenon has long been attributed to sensitivity to speech rhythm, the organized temporal structure of vowels and consonants (Mehler et al. 1988; Moon et al. 1993; Nazzi et al. 1998). While the relationship between early language discrimination and speech rhythm has never been explicitly tested, this presumed link has substantially impacted theories of language acquisition. Knowledge of the temporal regularity of one's native language is hypothesized to be acquired first and facilitate later learning such as word segmentation (Jusczyk et al. 1999) and syntactic structure, a theory called the prosodic bootstrapping hypothesis (Nazzi and Ramus 2003). Because sensitivity to speech rhythm forms the basis of theories of language acquisition, it is important to know whether infants are indeed sensitive to speech rhythm.

Despite the widespread hypothesis that newborns' language discrimination relies on sensitivity to rhythm in the speech stream, existing data are also compatible with other possible interpretations. Here we focus on the possibility that newborns' discrimination may instead reflect sensitivity to global and segmental acoustic properties of the speech signal. In contrast with rhythm, which must be inferred from the exact temporal sequence of the speech time series, global properties can be calculated by averaging short, segmental information without requiring information about any temporal order. It is already well known that there are global properties of speech that correlate with rhythmic classes, such as the mean percentage of vocalic durations (%V) and the variability of consonantal durations (Δ C) (Ramus et al. 1999). For the language pairs that infants were tested on, these are the only acoustic correlates that were directly compared with infant language discrimination. However, it is not yet known whether these global properties on their own would be sufficient to explain infants' discrimination, and most previous work that has measured these properties' correlation with rhythmic classes nevertheless assumes that what humans are sensitive to are the rhythmic properties, rather than the global properties (Ramus et al. 1999; Dominey and Ramus 2000; Langus et al. 2017).

In this paper, we show that early language discrimination can be simulated without using any rhythmic information. We conduct simulations of language discrimination using representations ranging from the acoustic level to higher‐level representations derived through several different models of speech perception, using intact speech as well as speech that has been scrambled to remove its rhythmic structure. We show that all of these representations across speech features and models can qualitatively simulate infant discrimination, implying that infants could be relying on global properties, rather than rhythm, in discriminating between languages. This has implications for theories of early language learning that center around rhythm and its crosslinguistic differences.

We first give background on the evidence in favor of infants' rhythmic sensitivity and existing challenges to this theory. We then give an overview of the framework and models we use for simulations. Our next sections present simulation results on natural and low‐pass filtered speech showing that a wide range of models and representations with or without access to rhythm can all capture newborn discrimination. Finally, we discuss the implications of these results for theories of language acquisition and their compatibility with other evidence on infants' sensitivity to rhythm.

## 2 Rhythm in Infant Language Acquisition

Infants as young as newborns are known to discriminate between certain languages but not others (Mehler et al. 1988; Nazzi et al. 1998; Ramus 2002). In these experiments, infants are exposed to utterances of one language, which is played through a speaker in the lab. When the speech stream switches to a new language, infants may notice the change, their response to which is reflected through increased sucking rate on a pacifier. A significant increase in sucking rate would indicate successful discrimination between two languages. It has been known that infants can discriminate between certain pairs such as English and Japanese, but not other pairs such as English and Dutch (Nazzi et al. 1998). The predominant explanation to date for the specificity in language discrimination is that discrimination occurs when the pair of languages differ in rhythm, the organized temporal structure of vowels and consonants in the speech stream (Ramus et al. 1999; Nazzi and Ramus 2003). Additionally, to ensure that the infants do not rely on segmental or lexical information in their discrimination, the speech stream in behavioral studies was manipulated through low‐pass filtering (Nazzi et al. 1998; Ramus 2002) or resynthesis (Ramus and Mehler 1999; Ramus 2002), where segmental information was replaced with synthesized sounds from similar categories (e.g., replaced any fricatives with /s/). These manipulations remove fine spectral information about phonetic categories and words, and to some extent control for the different phonemic inventory across languages. The persistence of language discrimination after such heavy processing indicates that infants are sensitive to what remains in the processed speech stream, which was presumed to be rhythm (Nazzi and Ramus 2003).

However, even in manipulated speech, language‐specific cues that are distinct from rhythm remain. As reviewed earlier, the two summary statistics that have been shown to correlate with human discrimination results, %V and Δ C (Ramus et al. 1999), were global statistics and can be calculated without access to the temporal sequence of segmental information. These global differences between languages likely stem from phonological properties related to timing, such as vowel duration (Ramus et al. 1999) and syllable structure (Langus et al. 2017). For example, more complex syllable structure in languages such as English can make some syllables much longer in duration than others compared with a language with simple syllable structure like Japanese. The complex syllable structure, in turn, can also affect the segmental acoustics due to consonant clusters (like “str”) that uniquely exist in languages with complex syllable structure. While these global properties may covary with the rhythm of a language to a great extent, it has been widely assumed in the literature that infants are sensitive to the temporal structure in the speech stream that generated the global statistics (rhythm), rather than to the global statistics themselves. These two signals carry similar information, but they have very different implications for understanding what cues in the speech signal infants attend to. In theories of acquisition, it has been hypothesized that later acquisition of, for example, word segmentation (Jusczyk et al. 1999) and phonetic categories in bilingual infants (Sundara and Scutellaro 2011) are based on sensitivity to rhythm, so understanding whether the sensitivity is to rhythm or to other global properties also has implications for understanding how infants bootstrap later learning.

Furthermore, in computational models that replicate infant language discrimination through simulations, it is also often concluded without controlled testing that the computational models succeed in perceiving “rhythm.” For example, in Dominey and Ramus (2000), a small connectionist model was trained to discriminate between annotated speech of various languages. The model's discrimination behavior aligns with that of newborns, which is taken as further evidence that human discrimination is also driven by local temporal regularity, even though no controls were included without access to temporal information. Additionally, in Carbajal et al. (2016), when a clustering‐based model showed language discrimination results on raw speech that aligned with what was predicted for humans, the model's success was attributed to learning of temporal regularities, which was made available to the model through feature engineering. In both cases, the computational models were assumed to use rhythm towards language discrimination, but this assumption was never tested.

While the prosodic bootstrapping hypothesis assumes that sensitivity to speech rhythm is a precursor to later language development, evidence from different fields has challenged how this occurs. Firstly, the knowledge of linguistic rhythm takes place at very different developmental stages across languages (Mazuka 2007). For example, while English‐learning babies demonstrated use of stress cues as early as 7.5 months (Jusczyk et al. 1999), the use of the mora as an overarching phonological unit does not appear in 4‐year‐old Japanese children (Kubozono 2000). This makes it unclear how the cues driving early language discrimination interact with language development. In production (Grabe et al. 1999; Payne et al. 2012; Polyanskaya and Ordin 2015), English‐learning children's pronunciation of English have rhythmic acoustic correlates that resemble that of Italian more than English, until they are 11 or 12 years old. This raises questions about whether acquisition of language‐specific rhythm really happens during the first few months of life, or is rather a lifelong process that takes until adulthood. Lastly, existing data are scarce about changes in language discrimination across development. As Gasparini et al. (2021) pointed out, among the only two studies that examined the effect of learning (Nazzi et al. 2000; Johnson and Braun 2011), the respective results have contradicting theoretical implications. Also, since both studies use naturalistic speech that was not manipulated, infants could discriminate between languages by relying on any combination of segmental (spectral and temporal cues) and suprasegmental (intonation and timing) information. As such, these empirical studies do not provide strong evidence that children are acquiring speech rhythm *per se*.

## 3 Simulating Early Language Discrimination

In our simulations, we model the qualitative results from infant language discrimination. We focus on discrimination of very young (3‐day‐old) infants (Nazzi et al. 1998), whose results inspired language discrimination studies on older infants as well as learning theories such as the prosodic bootstrapping hypothesis. In their experiment using a habituation paradigm, they found that 3‐day‐old French infants can discriminate between English and Japanese, but not between English and Dutch. Furthermore, in another experiment, they examined if the infants can discriminate between a mixture of languages switching to another mixture depending on whether the rhythm of the mixture is homogeneous. The authors found that discrimination was only possible when the group of languages was homogeneous in its “rhythm” characterizations.

Here, we simulate both studies using an array of computational cognitive models. In Simulation I, we simulate Experiments 1 and 2 in Nazzi et al. (1998), which tests language discrimination between a pair of languages. In Simulation II, we simulate Experiment 3 in Nazzi et al. (1998), which tests language discrimination between languages that are grouped by their rhythmic similarity.

### 3.1 Models

We examine three different models which generate different representations; we can interpret these as hypotheses regarding the cognitive representations used in language discrimination and early speech perception in general. These models range from deep learning embeddings to generative clustering algorithms. While each model represents a specific hypothesis about acquisition (e.g., predictive coding, generative inference, or adaptation), they have all been used in simulating human perception and early learning (Dominey and Ramus 2000; Vallabha et al. 2007; Feldman et al. 2013; Carbajal et al. 2016). As such, we simulate human language discrimination with all these models to highlight the generality of our findings.

Firstly, we used recurrent neural network (RNN) based on predictive coding, using a more modern architecture than Dominey and Ramus (2000). The RNN is a smaller version of the Vector‐Quantized Autoregressive Predictive Coding model (Chung et al. 2020), which predicts acoustic features in the future given current and past acoustic features. Our network contains three recurrent layers with vector‐quantizing (a bottleneck step to facilitate learning) after the last recurrent layer. For hyperparameters, the model we used contains 32 hidden units per layer and codebook size 16, and the network predicts nine frames in the future (i.e., 90 ms). The rest of the parameter choices are the same as Chung et al. (2020). We examine the same model trained for 10k, 100k, and 500k steps as well as the untrained model (i.e., random initialization only) to examine the effect of learning on language discrimination. In our experiments, we used embeddings from the first layer of the RNN as the model's internal representation on which language discrimination was tested (see Section *Test Procedure*).

Secondly, we used Gaussian Mixture Models (GMM), which cluster individual frames of the speech signals to find patterns. In cognitive science, similar models have been used to simulate early speech learning (Schatz et al. 2021; Li et al. 2020; Matusevych et al. 2023). This model assumes a “bag of speech frames,” meaning that speech frames are seen by the model in a way that is independent of their ordering, and the temporal structure therefore is not considered by the models. In our simulations, we trained models using scikit‐learn (Pedregosa et al. 2011) using 50 Gaussians with full covariance, initialized using k‐means and trained using Expectation Maximization until convergence (threshold $1\times 10^{-3}$). In theory, the model could be augmented to include timing information through feature engineering, but we did not perform that here.

Lastly, we used the i‐vector model following Carbajal et al. (2016). This can be seen as an extension of GMMs with an additional step: After a model is trained, it adapts to new data in a lower‐dimensional space, generating an adaptation (i.e., a shift from the original distribution) called an i‐vector. The i‐vector obtained this way can be used as the model's representation of this new utterance. Notably, in Carbajal et al. (2016), the i‐vector model was used directly to replicate language discrimination behavior in human infants. The model operates on engineered acoustic features: mel‐frequency cepstral coefficients (MFCCs) were computed from raw speech along with a pitch track, on which shifted delta coefficients (SDCs) were computed, allowing the features to contain information about speech over a longer time span (200 ms, as opposed to 10–30 ms in typical spectrogram‐based features), which is enough to contain suprasegmental information such as local syllable durations. The model behaved like human infants in discriminating languages across rhythmic classes (English and French) but not within (Spanish and Catalan). The success of the i‐vector model in these two studies was attributed to its access to longer temporal information over 200 ms. In our simulations, we replicate this i‐vector model with the original features containing suprasegmental and pitch information (“Original” model) on novel train and test corpora. Additionally, to test the claim that suprasegmental information led to human‐like language discrimination in the i‐vector models, we also simulated i‐vector models that cannot access slower temporal information by training and testing models with only segmental features. In one version, we only keep MFCCs in the feature set without temporal features or pitch track (“MFCC” model). In another version (“Spectrogram” model), to further remove spectral information, we extracted information using binned spectrograms similar to the other models. If suprasegmental and temporal information is necessary for language discrimination, we would expect the MFCC and Spectrogram models to not have humanlike language discrimination. We train all i‐vector models using the MSR Identity Toolkit (Sadjadi et al. 2013) with 256 clusters for the UBM, and a dimensionality of 200 for the i‐vectors, following Carbajal et al. (2016).

#### 3.1.1 Training and testing

We trained our models using French to simulate the language exposure of a newborn (3‐day‐old) infant.

For the GMM and i‐vector model, we trained each model using a small amount (1 h) of French obtained from the Globalphone corpus Schultz (2002), which contains roughly equal amounts of speech from four speakers (two males and two females).^1^ For GMMs, we trained and tested using two types of features, eight‐dimensional spectrograms as well as MFCCs (seven MFCCs and a pitch track). For i‐vector models, we trained and tested using four types of models detailed above to match the paradigms seen in the literature.

For the RNN model, since the type of self‐supervised deep learning algorithm is much more data hungry compared with other clustering models used in our simulations, we trained each model using 100‐h subsets (sampled randomly) from the Common Voice corpus (Ardila et al. 2019). While the Common Voice corpus was crowdsourced and contained a larger amount speakers with different recording conditions, it has enough data to train self‐supervised neural networks. Since the original RNN model (Chung et al. 2020), like other deep, self‐supervised speech models, is designed to operate on spectrogram features, we only trained the RNNs on log‐Mel spectrograms. We trained multiple models on disjoint training data to account for individual model differences—four models for the GMM and i‐vector model, and fivve models for the RNN.

In addition to the models above, we also performed testing using acoustic features, without any further modeling. For this, we use log‐Mel spectrograms as well as MFCCs as the representations of the signal and test them under the ABX task.

#### 3.1.2 Test procedure

We use the machine ABX task (Schatz et al. 2013; Schatz 2016) to evaluate language discrimination in machines. In the past, this technique has been applied to segmental categories (Dunbar et al. 2019; Schatz et al. 2021) and suprasegmental information such as language identity (Carbajal et al. 2016). In our case, we apply the ABX task to examine the models' ability to discriminate between languages. Behavioral experiments such as Nazzi et al. (1998) measured discrimination in newborns using a habituation‐disabituation paradigm. We choose the machine ABX task because it is an established way to probe model representations and compare models with human behavior. There is not a standardly accepted computational model of the specific behavioral paradigm used in this study (sucking, or habituation in general), and developing such a model has the drawback that it can introduce additional hyperparameters and degrees of freedom that may affect simulation results. The machine ABX task is a more conservative choice of evaluation method, with fewer degrees of freedom, and is meant to abstract away from any specific human behavioral paradigm.

The machine ABX task is conceptually similar to the human version of the ABX task, which is a 2AFC task where human receive three tokens, *A*, *B*, and *X*, and are asked to decide whether *X* is closer to *A* or *B*. In the machine, the decision of whether the machine chooses *A* or *B* depends on a distance measure calculated over the model's representations. The algorithm works as follows. In one trial, utterances *A*, *B*, and *X* are randomly sampled, where *A* and *X* are from one language (e.g., Japanese) and *B* is from the other (e.g., English). Then, the distances d(A,X) and d(B,X) are calculated. If d(A,X)<d(B,X), the machine is considered to be correct, since *X* is closer to *A* than *B* in the machine's representation. The choice of distance function depends on the representation of the model. For the GMMs, since the posteriogram representations are probability distributions, we used symmetrized Kullback–Leibler (KL) divergence. For all other model representations, we used cosine distance. For the i‐vector model, since only one i‐vector was generated for each utterance, the distance metric involves only the direct distance computation between the vectors illustrated above. For all other models, since the representations are generated for each time frame, we randomly sample one second^2^ of speech material from each utterance and perform dynamic time warping. Dynamic time warping finds the optimal temporal alignment between two segments. The use of dynamic time warping follows previous use cases of the machine ABX task (Li et al. 2020; Schatz et al. 2021) and allows features along temporal sequences to be compared frame‐by‐frame through a distance metric. In this case, we use dynamic time warping to align temporal structure in segments, such as syllable structure; in the scrambled condition, we also apply dynamic time warping to the sequence that no longer has temporal structure, for consistency. In our experiments, we used the sum instead of average of distances along the path generated by dynamic time warping, with a cost value of 1.

Since we have a large number of utterances, we randomly sampled *A*, *B*, and *X* in each trial and made 3000 independent draws for each model and condition, with each of the two languages being the correct answer half of the time (i.e., in 1500 draws). For example, to test the discrimination between English and Japanese, the ground truth of *X* would be English and Japanese for 1500 draws each. The ABX error rate was calculated over all the draws to represent the model's ability to discriminate between languages. A lower ABX error rate indicates better discrimination, and chance performance is at 50%.

### 3.2 Feature Preprocessing

All speech materials used in training and testing are mono and sampled at 16,000 Hz. From the speech waveform, we preprocess it in several ways matching the expected input for each model type based on the literature.

**FIGURE 1:**
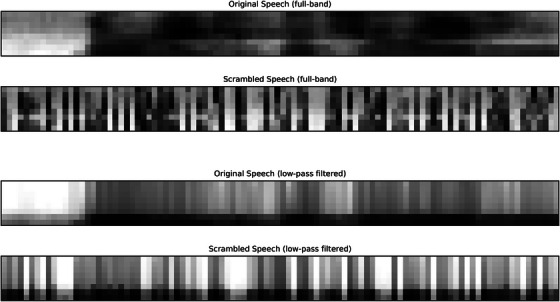
Examples of the log‐Mel spectrograms under two manipulations in our approach: original versus scrambling and full‐band versus low‐pass filtering. The spectrograms shown represent the first second of an English test utterance, which includes 100 time slices and 8 Mel frequency channels.

Spectrograms were used as input to each model and as acoustic features alone. From the speech waveform, we first perform short‐time Fourier transform (STFT) using Hamming windows with window length of 25 ms and hop length of 10 ms. The magnitude of the complex STFT spectrogram is taken to construct a magnitude spectrogram. Then, the linear frequency axis is banded on a Mel scale, and the magnitude converted to logarithm to create the log‐Mel spectrogram. Lastly, the log‐Mel values are normalized using *z*‐score normalization on a per‐utterance basis. For simplicity and to remove most of the spectral contents, we used only eight frequency channels on a logarithmic scale.^3^ The resulting low frequency resolution removed detailed information such as harmonics and formants, which obscures detailed spectral information such as vowel identity and pitch. An example of the resulting spectrogram is shown on the top of Figure 1. The spectrogram contains information about the short‐time spectrum of speech along time. Since we only use eight frequency channels, the spectral information contained in the spectrogram is degraded to only contain coarse details, making fine spectral differences such as different vowels less distinct, but still highlights coarse differences such as vowels versus consonants.

For the GMM, i‐vector models, and acoustic features, we also used MFCC features following Carbajal et al. (2016), using window length of 25 ms, hop length of 10 ms, and retaining the first seven MFCCs. Additionally, a F0 track was added using the YIN pitch estimator. Specific to the i‐vector model, the resulting eight‐dimensional features are also augmented with SDCs using a 7‐1‐3‐7 configuration resulting in 64‐dimensional features per time slice that contains information spanning approximately 200 ms. The MFCC features contain information about both coarse and fine spectral properties, and in the case of SDCs, also contain slower temporal information.

For each model and feature type, we also perform two manipulations, which we introduce below.

#### 3.2.1 Temporal scrambling

We temporally scramble (or shuffle) the segmental frames in our test stimuli to examine whether language discrimination is driven by rhythm. Since the speech signal is transmitted in the time domain, any information in speech is characterized through temporal regularities. Among all temporal regularities, the faster ones correspond to segmental information such as formant and harmonics, and the slower ones correspond to syllable, word, and phrase‐level rhythm. In our study, we focus on the effect of slower regularities, which we reflect through the ordering of segmental information, such as the order of individual frames of a spectrogram. Since the spectrogram frames are 10 ms apart from each other, any temporal information that is slower than 100 Hz is included in this range, including syllable rate (4–8 Hz) and slower regularities such as stress and prosodic phrase (Poeppel and Assaneo 2020). To remove the slower rhythmic information, we randomly shuffled the acoustic features to disrupt any temporal structure that is slower than 10 ms in the speech signal. An example of a scrambled utterance is shown in Figure 1. We introduce this condition to remove temporal information either directly used by the model (i.e., the RNN and the Full version of the i‐vector model) or used during the test procedure through dynamic time warping (i.e., in the RNN and GMM).

#### 3.2.2 Low‐pass filtering

In behavioral studies, the speech stimuli were often low‐pass filtered to remove finer spectral information such as formants, often as a means to rule out the confounds of acoustic difference between languages (such as having different vowel inventories) between the language in the observed language discrimination behavior (Nazzi and Ramus 2003). In all our simulations, we run tests on test stimuli that are unfiltered (“raw”) as well as low‐pass filtered. For low‐pass filtering, we choose a cutoff frequency of 400 Hz following the setup in Nazzi et al. (1998), implemented as a fourth‐order Butterworth filter.

### 3.3 Simulation I: Discrimination of Language Pairs

In the first set of simulations, we simulate language discrimination between two languages. Following the first two experiments in Nazzi et al. (1998), we tested English–Japanese and English–Dutch. In the behavioral study, 3‐day‐old infants dishabituated at a switch between English and Japanese, but not between English and Dutch. In this simulation, we replicate this effect in models that can or cannot access rhythmic information.

**FIGURE 2:**
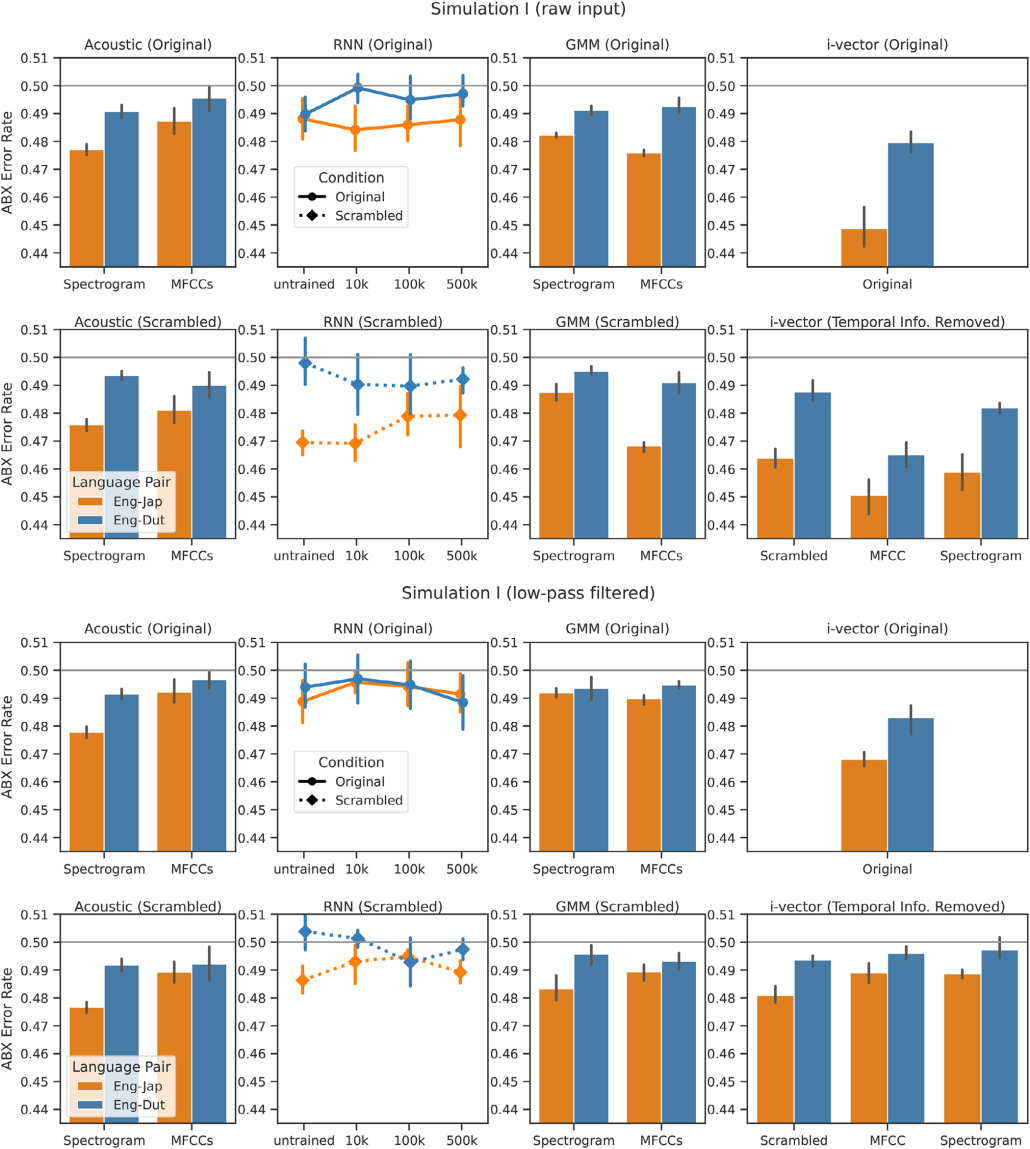
Language discrimination results on single languages. Within full‐band and low‐pass filtered stimuli, the top row groups results on stimuli with temporal order, and the bottom row groups results without temporal information. A lower ABX error rate indicates better discrimination, where chance level (50%) is marked in gray. Errorbars represent 95% CI calculated over models trained on disjoint training data. For the acoustic representations, since no models were trained, we performed five statistically independent tests over which the error bars were calculated.

To test the models, we used utterances from the Common Voice corpus (v. 13.0) (Ardila et al. 2019). Among all sentences, we discarded sentences with downvotes (i.e., rated by online participants to have noise, dis‐fluency, or bad otherwise; around 20%). For each set, we selected utterances that are between 4 and 10 s long. The utterances were shuffled to avoid selecting multiple clips from the same speaker. Then, we manually listened to the audio files and selected the first 100 utterances per language without significant noise, disfluencies, or obvious non‐native accents. Through the manual selection process, the criteria filtered out roughly 50% of the utterances. The IDs of the selected utterances are included in the code release at https://github.com/smiledra/early‐language‐discrimination‐rhythm. All utterances were root‐mean‐square normalized before any further processing. The results are shown in Figure 2.

Results showed significantly better discrimination on English–Japanese than English–Dutch in all models and all representations, indicating that the models' performance replicates that of human infants in Nazzi et al. (1998). This is true for both intact speech and temporally scrambled speech, indicating that rhythm is not important to the effect of language discrimination. For the i‐vector models, the crosslinguistic effect was observed not only in the Full model which contains slower temporal features, but also in the Scrambled model where temporal information was shuffled, as well as the MFCC and Spectrogram models where slower temporal information was unavailable. This indicates that language discrimination can be achieved using segmental information alone, without using slower temporal regularities that are crucial for rhythm.

In certain cases (e.g., in the case of some RNN models and the GMMs when stimuli were low‐pass filtered), scrambling in fact led to stronger humanlike results. This result seems counter‐intuitive for two reasons. First, for both models, dynamic time warping at test time must align the test utterances, which could be harder if the stimuli are scrambled and lack syllabic and phrasal structures for alignment. Secondly, for the RNN model, since the model is sensitive to the temporal sequence due to its recurrent structure, the temporally scrambled stimuli are out‐of‐distribution compared with the training data. However, given our hypothesis that language discrimination can be achieved by relying on global information (e.g., %V), temporally scrambling the frames would lead to a more even distribution of global information. This would make the global statistics more consistent when computed over different stretches of speech, leading the *A* and *X* stimuli in the ABX task to be more reliably similar. Therefore, the increase in effect size after scrambling the stimuli serves as evidence for our hypothesis that language discrimination in these models may rely on global properties rather than temporal structure.

Additionally, human‐like language discrimination can be achieved with both scrambled and unscrambled acoustic representations, especially in the spectrogram case. As spectral information is highly degraded in these spectrograms with only eight spectral dimensions, cues requiring fine frequency details (e.g., formants and frication centroids) are absent. However, global information such as the distribution of vowels and consonants should remain intact. This again suggests that discrimination is possible using global information alone, even based on acoustics without further processing. We also observed slightly stronger effect from the spectrogram features compared with MFCCs in the acoustics case. Since seven‐dimensional MFCCs retains much more detailed information about coarse and finer spectral information along with the extra pitch track, it is possible that the removal of detailed information allows the model to solely rely on global information, which makes discrimination easier for, for example, English–Japanese.

#### 3.3.1 Discussion

In discriminating language pairs, the models tested were generally successful in replicating human‐like results. Specifically, among all the model conditions that displayed human‐like effect in temporally ordered speech, they all displayed an effect in scrambled speech. This suggests that temporal regularities are not necessary for language discrimination in these models.

However, when discriminating low‐pass filtered speech, some models showed mixed (in the GMM) or null (in the RNN model) effects. Considering that low‐pass filtered speech (in test) was greatly different from unfiltered speech (in training), especially in that the higher‐frequency channels were set to zero after low‐pass filtering, the distributional shift between training and test data might lead to greater difficulty to obtain any meaningful effect from these models. For example, in the RNNs, the embeddings of the test utterances could generally be distant from each other regardless of language, thus leading to discrimination scores that are not crosslinguistically different. Human infants, on the other hand, are not subject to such numerical constraints (Nazzi et al. 1998). This may be due to the inherently different implementation of human perception and cognition, which relies on networks of neurons rather than computer bits, or the prenatal exposure to speech which has some low‐pass characteristics (Griffiths et al. 1994). Nonetheless, even under numerical difficulty, temporal scrambling seemed to help the model behave like human infants, which cannot be explained if temporal regularities are necessary for human‐like language discrimination.

### 3.4 Simulation II: Discrimination of Mixed Languages

**FIGURE 3:**
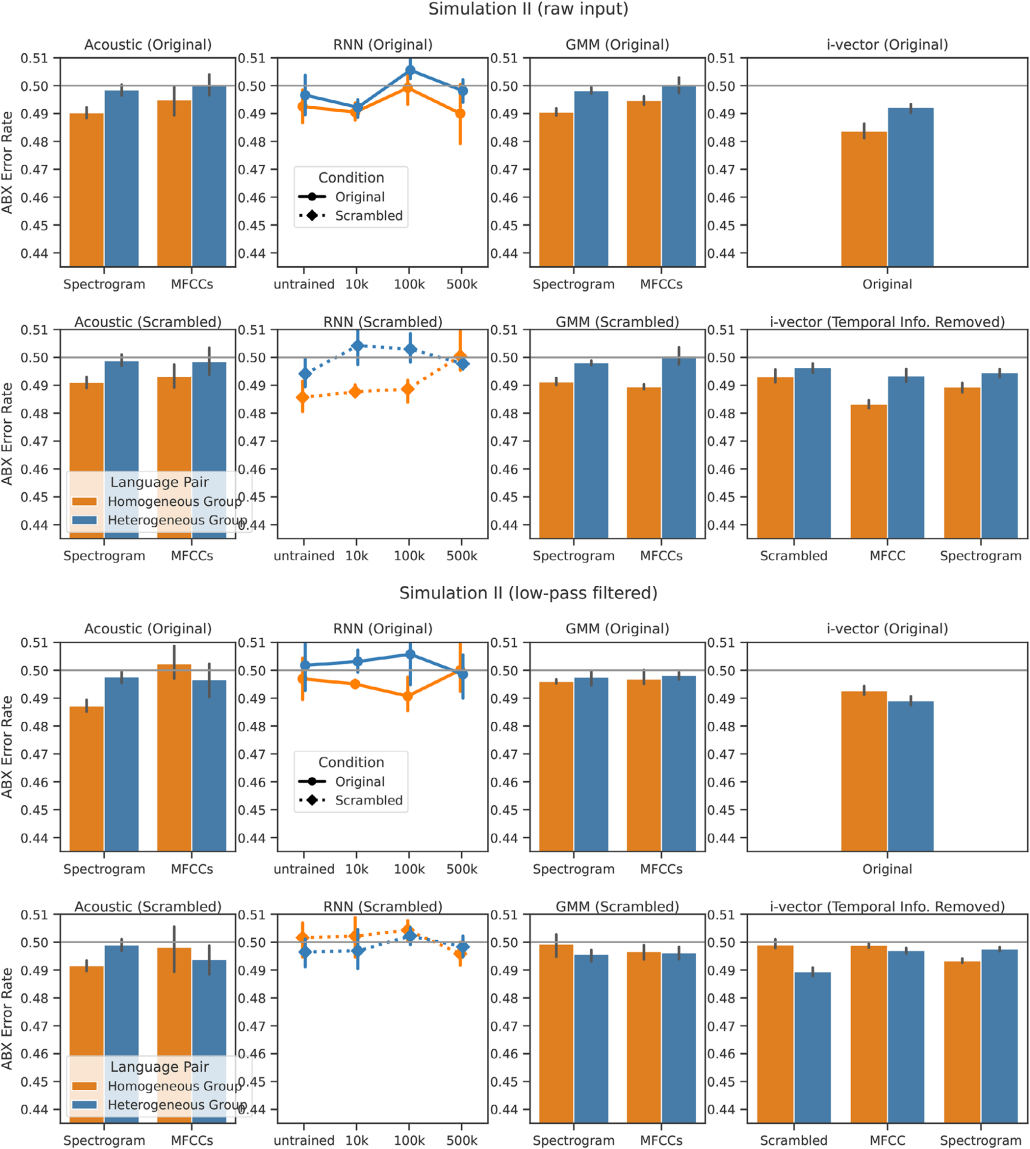
Language discrimination results on grouped languages. Within full‐band and low‐pass filtered stimuli, the top row groups results on stimuli with temporal order, and the bottom row groups results without temporal information. A lower ABX error rate indicates better discrimination, where chance level (50%) is marked in gray. Errorbars represent 95% CI calculated over models trained on disjoint training data. For the acoustic representations, since no models were trained, we performed five statistically independent tests over which the error bars were calculated.

In the first set of simulations, we tested the model on discriminating between language pairs, and found the results to replicate language discrimination in human infants. However, discrimination between two single languages can be confounded with factors specific to the languages chosen (sound inventory, language‐specific prosody, etc.) and the data collection process of each corpus (recording device, background noise, etc.). In the second set of simulations, we model Experiment 3 in Nazzi et al. (1998) to test the discrimination between two groups of languages. The original study found infants to dishabituate more when languages switch across a homogeneous group (i.e., from a group containing English and Dutch to a group containing Spanish and Italian, vice versa) than across a heterogeneous group (i.e., English and Spanish vs. Dutch and Italian). We simulate language discrimination using computational models and test whether the models replicate human behavior. We test two pairs of groups: in the homogeneous group, we grouped English with Dutch, which switches into a group containing Spanish and Italian (and vice versa); in the heterogeneous group, we grouped English with Spanish, which switches into a group containing Dutch and Italian. Since all the utterances used in the two conditions are the same, only switching between different sets, this can eliminate some confounds specific to the speech material. We used the same corpus and selection criteria for test stimuli as Simulation I. Similar to Simulation I, speech segments are sampled from a group of utterances for the ABX task, except that the set of utterances from which the test stimuli are sampled consists of a group of languages in the current simulation, instead of a single language. The results are shown in Figure 3.

When the test stimuli were unfiltered, language discrimination for the models mostly aligned with that of humans, which is similar to Simulation I. For the Acoustic model and the GMM, the significant effect remained regardless of scrambling. In the RNN model, with unscrambled stimuli, language discrimination was in the human‐like direction although insignificant. With scrambling, the results were significant in the RNN across all three training amounts. This is similar to the RNN's behavior in Simulation I: When the test stimuli was scrambled to become temporally uniform, the effect size increased, possibly because global information helped with discrimination more than temporal regularity. In the i‐vector model, we also observed human‐like discrimination in the Full and MFCC model, suggesting that temporal regularity was not necessary for human‐like behavior. The Full Scrambled model, however, showed a marginal yet insignificant outcome. Instead, in the MFCC and Spectrogram models, when temporal information was removed altogether directly, discrimination aligned with human behavior.

When the stimuli were low‐pass filtered, the results are less consistent across models. In the acoustic spectrograms and the spectrogram version of the i‐vector model, however, the results were human‐like. For the other models and features, however, the crosslinguistic effect was inconsistent regardless of the availability of temporal information in the input features.

From the second set of simulations, we conclude that different models were generally able to discriminate the rhythmic group better than the nonrhythmic group, even when temporal structure is inaccessible to the model. However, when the test stimuli were low‐pass filtered, some of the models failed to replicate human behavior completely, while the successful models were more likely to replicate human behavior with the absence of temporal regularities. Overall, in no case, do we see a model consistently succeed with temporal information available, and consistently fail without it.

## 4 General Discussion

We used computational models to simulate language discrimination using naturalistic speech as well as manipulated conditions to remove temporal regularities. We find various models to be able to replicate the behavioral discrimination pattern, even when temporal regularities are not accessible to the models. This suggests that language discrimination can be achieved through information other than temporal regularities, a result that calls for further investigation into the nature of language discrimination in human infants.

Our results have implications for theories of language development that centered on the role of speech rhythm in early acquisition. First of all, it directly challenges the prosodic bootstrapping hypothesis (Nazzi and Ramus 2003), which proposes that an early sensitivity of rhythm drives acquisition of metric units in one's native language. The prosodic bootstrapping hypothesis specifically assumes that sensitivity to speech rhythm narrows during the first year of life, which our results challenge directly by calling into question whether speech rhythm plays a role at all during newborn language discrimination. Our results also challenge the theory in bilingual acquisition that rhythmic similarity between the languages being learned is what affects the timing of vowel discrimination and word learning (Sundara and Scutellaro 2011). While languages that are considered “rhythmically similar” are more similar in acoustic correlates of speech rhythm, they share other global and segmental properties compared with “rhythmically dissimilar” languages, which the current study supports as likely causes of language discrimination. Lastly, our findings can potentially influence the narrative of language development, which currently emphasizes speech rhythm as being among the earliest available cues to infants that drive further language development (e.g., Werker and Hensch 2015; Gervain et al. 2020).

In our simulations, one major difference between our test paradigm and the behavioral experiment in Nazzi et al. (1998) was the difference in the distribution of test speakers. In behavioral studies like Nazzi et al. (1998), the test stimuli were obtained from two female speakers with minimal voice quality difference between each speaker. On one hand, factors such as voice quality are subjective and difficult to replicate. On the other hand, additional variability such as language background (Lin and Wang 2008; Li and Post 2014) and age (Pellegrino et al. 2021), are not typically reported in behavioral studies but are known to affect speech rhythm. To address these sources of variability, in our simulations, we used utterances from a wide range of speakers selected from crowd‐sourced corpora. While this introduces additional noise in the test stimuli, thereby making the discrimination task harder, we still observe significant effects of language pair on discrimination, suggesting that we successfully replicated the language discrimination phenomena while ruling out confound variables noted above.

One difference between our results and the behavioral studies is the absolute existence of an effect. In Nazzi et al. (1998), infants showed an absolute inability to discriminate between English and Dutch, while being able to discriminate between heterogeneous language groups. In our simulations, many models did not follow this exact pattern, with some models showing chance‐level discrimination for heterogeneous language groups, or significantly above chance for English–Dutch. We attribute this to two fundamental differences between the human study and our simulations. First, while the machine ABX error rate can be different with any detected differences between the two languages tested, human perception may require certain differences to reach certain thresholds before showing a behavioral response. Therefore, the machine ABX results could be oversensitive and their absolute values less useful than relative comparisons, such as English–Japanese against English–Dutch. Second, while the machine ABX task uses three utterances per trial, infants are able to hear many more utterances through the habituation–disabituation paradigm such that they may be able to bootstrap in‐group similarity better than the machine, allowing them to detect a difference between heterogenerous rhythm groups while the computational models failed to do so. Both explanations suggest that rather than the absolute value of scores, a better comparison between behavioral data and simulation results is to compare relative scores in a controlled setting, which is how we analyzed the results.

### 4.1 Language Discrimination With Altered Segmental Properties

In past studies on the behavioral and neural response in young infants to speech rhythm, various manipulations on the speech stimuli have been used. The fact that language discrimination remains despite removal of some segmental properties (such as low‐pass filtering and resynthesizing) but not when speech is temporally manipulated (such as playing speech backwards) has been traditionally taken to establish infants' sensitivity to rhythmic cues in speech. Here, we argue against the claim that these baselines provide evidence of rhythm sensitivity per se.

In behavioral studies about newborn language discrimination, stimuli were often low‐pass filtered to remove information at higher frequencies. Since lowpass filtering (e.g., at 400 Hz) removes spectral information such as most formants and high‐frequency consonants such as sibilants, it is assumed that lowpass filtering removes phonetic and phonotactic information but retains prosodic (and as a subset of prosody, rhythm) information. These assumptions motivate behavioral studies such as Nazzi et al. (1998) to low‐pass filter the stimuli as a way to retain prosodic information while greatly removing spectral information. The fact that infants were able to discriminate between rhythmically different languages after lowpass filtering has been taken as evidence that they used suprasegmental, and likely rhythmic, cues for this discrimination task.

However, lowpass filtering speech is not well‐justified to extract rhythm, and the exact purpose of using lowpass filtered stimuli to examine rhythm perception remains ambiguous. While lowpass filtering removes segmental information, it retains information like pitch contour, which is known to be a factor that contributes to human language discrimination (Chong et al. 2018). Additionally, since the cutoff frequency of 400 Hz may be higher than the first formant of some vowels and different numbers of harmonics depending on the speakers' pitch, the exact content being removed and retained from the speech stream is unclear. As a result, low‐pass filtering may not be the best way to test for rhythm sensitivity.

Another early motivation for using lowpass filtered speech to test language discrimination is that lowpass filtered speech may resemble in utero exposure to speech more than fullband speech, and infants therefore may use certain knowledge from prenatal speech exposure towards the language discrimination task (Mehler et al. 1988). While it is true that higher frequencies are attenuated through the maternal body and amniotic fluid, a significant level of noise has also been found in the fetal environment, likely due to maternal heart and other organs (Abrams et al. 1998). As measured in a pregnant ewe, this noise can reach 60–80 dB SPL, and is highest in lower frequencies of <100 Hz. The loudness and low‐frequency characteristics make low‐pass filtered full‐band speech an unsuitable representation of the human fetal sound exposure. Additionally, Hepper and Shahidullah (1994) tested human fetuses' response to pure‐tone stimuli at different frequencies. They found that a developing fetus responds to mid‐range frequencies (500 Hz) first at 19 weeks of gestation, and gradually expands to a wider band of 100–3000 Hz when they are close to full‐term. This gradual change of fetal auditory frequency selectivity likely stems from the prenatal maturation of the fetal auditory system, as well as the size and position of the fetus. Overall, these findings indicate that stimuli of lowpass filtered speech at 400 Hz, which was used in the earlier behavioral studies, are not ideal in representing prenatal exposure.

Another manipulation to speech was to resynthesize the speech to remove confounding segmental or prosodic information. In Ramus and Mehler (1999), French adults were able to discriminate between English and Japanese when speech was resynthesized to “SASASA” with pitch contour removed, but not when speech was resynthesized to “AAAA” with original pitch contour. The only two differences between these two conditions are the removal of sibilants, which are high‐frequency, and the addition of pitch contours, which is available in lowpass filtered speech. However, for the “AAAA” condition, which is more similar to lowpass filtered speech, discrimination between English and Japanese was not achieved. This suggests that lowpass filtered stimuli, which is conceptually similar to the “AAAA” manipulation, may not be the best manipulation for the observed language discrimination effect. On the other hand, the eight‐channel spectrogram in our study greatly reduced spectral information such as pitch and formants, but retains the difference of, fror example, vowels and sibilants, and is more conceptually similar to the “SASASA” manipulation. Additionally, it is shown that pitch contour may play a role in infant language discrimination as well. In Chong et al. (2018), 7‐month‐old infants' discrimination between English and German, two languages that are similar in rhythm, was attributed to sensitivity to different intonation (i.e., pitch contour) between the two languages. These results indicate that the perception and development of rhythm may be separate from intonation or pitch contour, where lowpass filtering is not a technique that can tease apart the two. In addition to behavioral studies, Tilsen and Arvaniti (2013) used a data‐driven approach to analyze the envelope of various languages and found correlates of syllable‐level and stress‐level rhythm, but critically, speech envelopes in this study were obtained by high‐pass filtering at 400 Hz to remove the influence of energy from the fundamental frequency on the envelope. This further calls into question whether frequencies below 400 Hz serve as a useful cue to speech rhythm, or rather contains separate information such as pitch.

To replicate behavioral studies, we tested our models with both unfiltered and low‐pass filtered speech, where models showed weaker cross‐linguistic effects on low‐pass filtered stimuli. In addition to the arguments above that cast doubt on the validity of using lowpass filtered speech to examine language discrimination and rhythm, we also note some more constraints specific to the nature of the computational models used. In generative models (which GMMs and i‐vector models belong to), stimuli are assumed to be generated from hidden distributions, where the disparity between training and test stimuli can limit interpretation or even cause numerical issues. As a result, training on fullband speech and testing on low‐pass filtered speech is difficult for such models. On the other hand, newborn infants already received in‐utero exposure to speech with attenuated high frequencies, which may allow meaningful information to be extracted and compared from lowpass filtered speech. Due to these difference between machines and humans, we expect the models to perform in a more noisy way with lowpass filtered stimuli. As a future direction, it would help us understand the gap between the perception of human infants and machines (cf. Vogelsang et al. 2023).

Some behavioral studies also use backward speech to show that the temporal structure of real speech is important for discrimination, and find that infants are sensitive to cross‐linguistic differences in rhythm in forward speech but not backward speech (Mehler et al. 1988). Backward speech would be identical to forward speech in terms of acoustic correlates of rhythm (e.g., %V, Δ C, amplitude modulation), so if infants are sensitive only to those correlates, then they should retain their ability to discriminate between rhythmically different languages when speech is played backward. Indeed, for our models, backward speech produces similar results to forward speech, which we report in the supplementary materials. This model behavior does not align with the behavioral data from infants. However, it is possible that infants' failure to discriminate backward speech stems from the fact that backward speech is out of distribution for infants, similar to how lowpass filtered speech is out of distribution for the computational models in question. Since backward speech is not an ecologically valid listening experience, infants may not be able to extract the segmental or suprasegmental cues in the same way as when they listen to naturalistic speech.

We might predict that if our temporally scrambled stimuli were able to be reconstructed in the time domain and played to humans, they would likely sound jarring and listeners might find it difficult to extract any meaningful information for making discrimination judgments, similar to the backward speech. However, conceptually similar results to our scrambling manipulation have been observed in visual language discrimination. In visual language discrimination (Weikum et al. 2007), infants were tested on their ability to discriminate between different languages, but from silent video recordings of the speaker instead of the auditory speech stream. In follow‐up work that has not yet been published, the visual recording was cut into chunks of 200 ms and the chunks were scrambled randomly, thereby removing any information slower than this timescale. While 4‐month‐old infants successfully discriminated between scrambled English and French, 8‐month‐olds failed to do so (Weikum et al. unpublished manuscript). This offers behavioral evidence to support the hypothesis that younger infants are able to discriminate between languages with the removal of rhythm to some degree. Older infants' discrimination is conditioned on intact slower rhythm, which suggests a change in the importance of rhythm across development. This aligns with our findings and further challenges the theory that rhythm sensitivity precedes other cues in language development.

To summarize, although various manipulations have been tested in behavioral studies, in comparing human and machine behavior, it is necessary to consider the ecological validity of the stimuli for both humans and machines. While our models were less consistent in simulating infants' language discrimination when tested on low‐pass filtered speech than on unfiltered speech, we have argued in this section that when considered together, experimental controls such as low‐pass filtering that have removed various segmental properties from speech still do not provide strong evidence that infants are attending to rhythm in tests of language discrimination.

### 4.2 Relation to Other Literature

**FIGURE 4:**
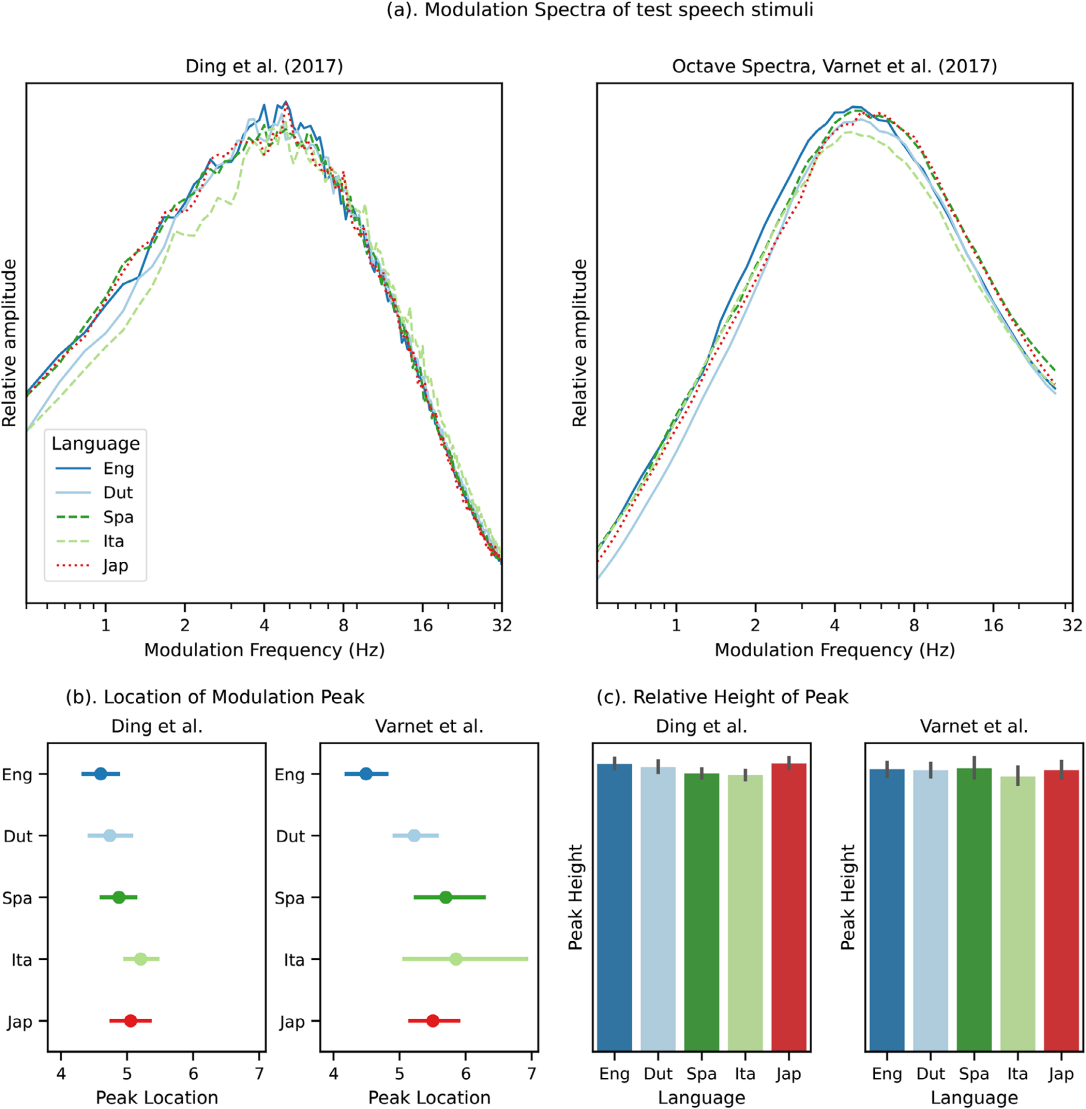
(a) Amplitude modulation spectra of the test stimuli used in the current study, using the methods from Ding et al. (2017) (left) and the octave spectra method in Varnet et al. (2017) (right). (b) Peak locations of the modulation spectra for each test language. Dots represent average over all utterances, and the error bars denote 95% CI. (c) Relative height of the peaks. Bars represent average over all utterances, and the error bars denote 95% CI.

While newborn infants may be using cues other than rhythm in language discrimination, we also examine the relationship between our stimuli and slow amplitude modulations, which have been described to reflect rhythmic differences. Specifically, all spoken languages have been found to have amplitude modulation peaks around 4.6 Hz (Ding et al. 2017), where the specific location and height of the modulation spectrum peak was correlated with rhythmic similarity (Varnet et al. 2017). In theory, the location and height of the peak would respectively reflect the average syllable rate and the variability around the average, which would be greater for languages such as English and Dutch. However, evidence exists against the robustness of using such measures (Zhang et al. 2023). Namely, the correlation between language rhythm and measurements of the modulation spectrum is not robust to varied speaking style and less than several minutes of speech. In our simulations, we also analyzed the amplitude modulations using the approaches from both Ding et al. (2017) and Varnet et al. (2017) (Figure 4). We discovered little difference between all five languages in terms of the location and height of the peak, and the variability in the location and height of the peak is not organized by rhythmic typology. Therefore, for the current set of stimuli, while our models are able to replicate human perception, the amplitude modulation spectra do not correlate with rhythmic similarity. We therefore present a case where languages did not differ in their temporal modulation at the syllable rate, but are nonetheless discriminable by models tested in this study, many of which do not have access to any temporal regularities.

In the cognitive neuroscience literature, there are a few related studies that explored crosslinguistic differences in early language perception through neural recordings. Our simulation results are compatible with their observations. For example, during passive listening, infants' EEG activity was shown to be different in gamma band when listening to different languages (Peña et al. 2010), which corresponds to the segmental (phoneme and subphoneme) level of information. Meanwhile, no difference was found in slower modulations that correspond to syllable‐level rhythm, such as the delta and theta bands (Giraud and Poeppel 2012). In other passive listening studies to multiple languages, while both evoked and phase‐based analyses were performed on the EEG data, only evoked responses were found to be different across languages, while the phase‐based analyses stayed the same (Nacar Garcia et al. 2018; Barajas et al. 2021). Based on the presumed connection between rhythm and amplitude modulation (Varnet et al. 2017), differential markers should be expected in frequency‐domain analyses instead of evoked responses, since the presumed amplitude modulation difference across languages were found in the frequency domain, and a significant evoked relationship can indicate anything in the speech stream, including anything prosodic or segmental. Nonetheless, neural recording studies that are more closely related to language discrimination are needed to arrive at stronger conclusions.

While our experiments call into question whether rhythm is the driving factor for language discrimination, they do not challenge whether infants are sensitive to rhythm at all. In fact, infants are sensitive to pure‐tone rhythm patterns subject to the iambic‐trochaic law even at birth (Abboub et al. 2016). Unlike naturalistic speech which is complex and contains many confounding factors, the controlled pure‐tone stimuli only differ in their temporal order such that scrambling removes the difference between an iamb and a trochee. Therefore, infants' behavioral effect must arise from actual sensitivity to temporal regularity. However, the specific connection between the observed effect on pure tones and older infants' ability to segment words from the speech stream (Jusczyk et al. 1999; Polka and Sundara 2012) should be considered more carefully.

## 5 Conclusion

This paper has argued that newborns' ability to discriminate rhythmically different languages does not provide strong evidence of their sensitivity to rhythm. Our simulations showed that a wide variety of models that can predict the relative ease with which newborns discriminate languages can still do so when rhythmic information was eliminated from the stimuli. We then argued that across various manipulations with intact and low‐pass filtered speech, global properties such as the percentage of vowels are equally accessible and can be driving the discrimination behavior. As the only direct acoustic correlate that is directly compared with human performance contain these summary statistics that are only related to rhythm in theory (Ramus et al. 1999), existing evidence about rhythm being important in early language discrimination is not strong enough. Lastly, we discussed our results with respect to amplitude modulation, a measure of temporal structure in speech that has been found to correlate with rhythmic classes of languages, and found that it is unlikely to be available as a cue in the types of tasks that are used with infants. Together, these results call for a reconsideration of the nature of language discrimination in human infants.

As theories of language acquisition often center around rhythm, our results push the field towards a reconsideration of the role of rhythm in early language acquisition. Specifically, speech rhythm has been considered as one of the earliest accessible cue to infants, and presumably learned by the infants prior to learning segmental and lexical information. As our results suggest that speech rhythm may not be relevant to language discrimination, or even accessible to infants, the role of rhythm in language acquisition may be different than what has previously been supposed.

## Conflicts of Interest

The authors declare no conflicts of interest.

## Supporting information




**Supporting File 1**: desc70220‐sup‐0001‐SuppMat.pdf

## Data Availability

The code for our computational simulations is available on GitHub. Along with the code, we also released the IDs to the curated test stimuli set, such that the test stimuli can be re‐extracted from public datasets for replications.

## Endnotes

1 The 1 h of speech was selected by choosing four individual speakers from the Globalphone corpus with approximately 15 min of speech material, which is close to the mean per‐speaker speech material in this corpus. All of the speech from those four speakers was used for training.

2 We also ran tests with 0.5 and 2 s of speech material, with qualitatively similar results.

3 Replication using 80 frequency channels showed qualitatively similar results.
