# Supplementary material for: Newborns' Language Discrimination May Not Reflect Sensitivity to Speech Rhythm: Evidence From Computational Modeling
Source: Dev Sci. 2026 May 8;29:e70220. doi: 10.1111/desc.70220 (PMC13155068; doi:10.1111/desc.70220)
Supplement: Supplementary file 1 — Supporting File 1: desc70220‐sup‐0001‐SuppMat.pdf [file DESC-29-e70220-s001.pdf]

Supplementary Material

1 Additional simulation: backward speech

In the main text, we have shown simulations on original speech stimuli with rhythm preserved as well simulations with temporal information removed by scrambling the order of short-time frames. In behavioral studies, speech is often also played backward as a means to remove linguistic information (such as lexical recognition) but still retain temporal order to some extent. In the context of our simulations, this manipulation is relevant to some of our models and feature types. For the RNNs, as the models were trained to predict upcoming speech information, testing on backward speech could diminish the language discrimination results as RNNs may generate less interpretable outcome when made to predict upcoming speech information on backward speech. Similarly, for the full version of the i-vector model, since it is trained with slow time information of up to 200 ms, backward speech may differ in the distribution of these features. For all other models, since the models themselves do not receive short-time speech input in a particular temporal order, and that dynamic time warping in evaluation is not directional itself, meaning that it would provide the same alignments for forward and backward speech, we do not expect these models to differ in their results for backward speech.

We performed additional simulations for the RNN model and the full version of the i-vector model. The feature preparation step and evaluation setting are identical to the main text, except for the test stimuli being reversed in temporal order at the spectrogram stage.

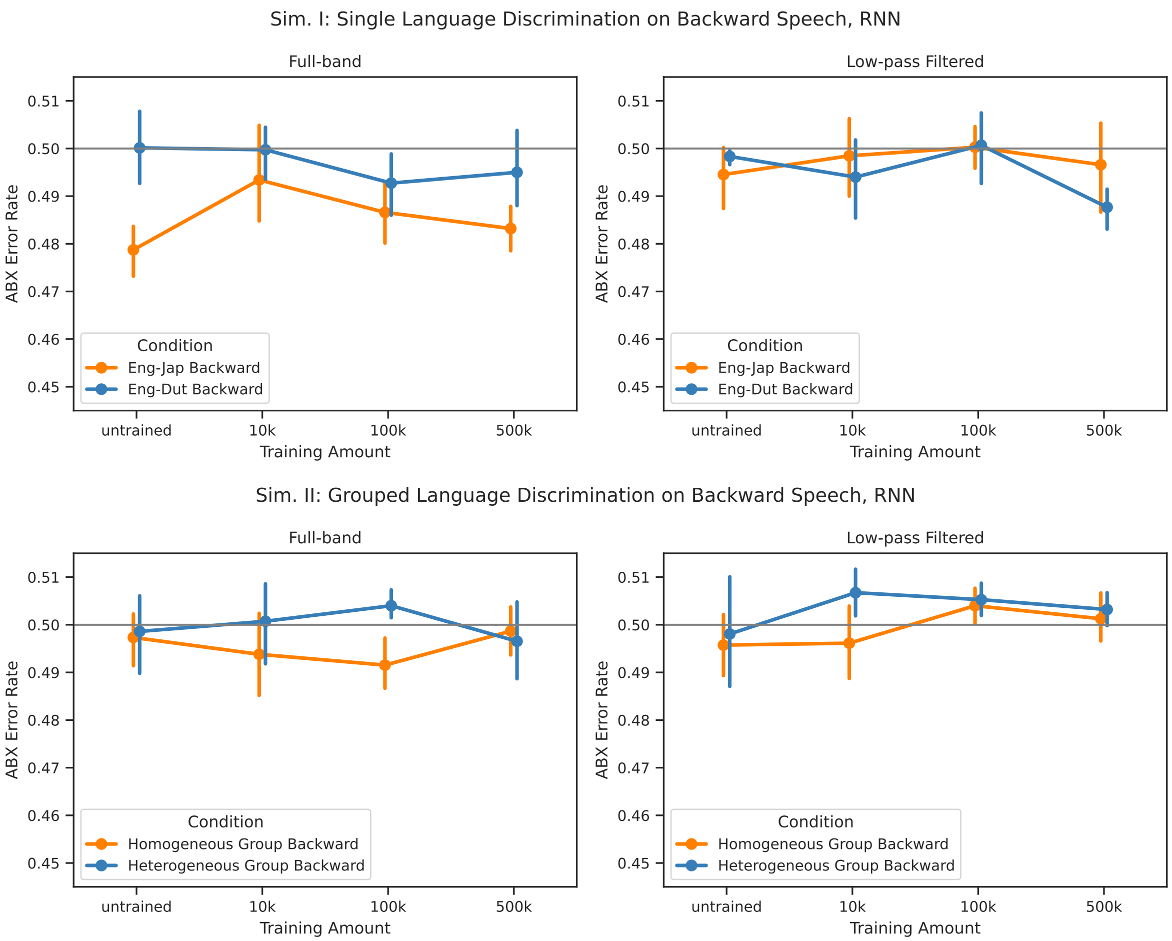

Figure 1: Backward speech results on the RNNs. A lower ABX error rate indicates better discrimination, where chance level (50%) is marked in grey. Errorbars represent 95% CI calculated over models trained on disjoint training data.

Since spectrograms are created from the speech waveform via fast Fourier transform, which yields identical spectrum for forward and backward waveform, this is identical to reversing the waveform in the behavioral studies.

In results for the RNNs (Figure 1 and i-vectors (Figure 2, it can be observed that the effects of language discrimination observed are very similar to the results on forward speech in the main text. For the RNN, in both backward and forward speech, the effect size is not significant in most cases, which contrasts with scrambled speech, where the humanlike effect is much larger. This further supports our hypothesis that scrambling speech contains more uniform segmental and global information, which contributes to humanlike language

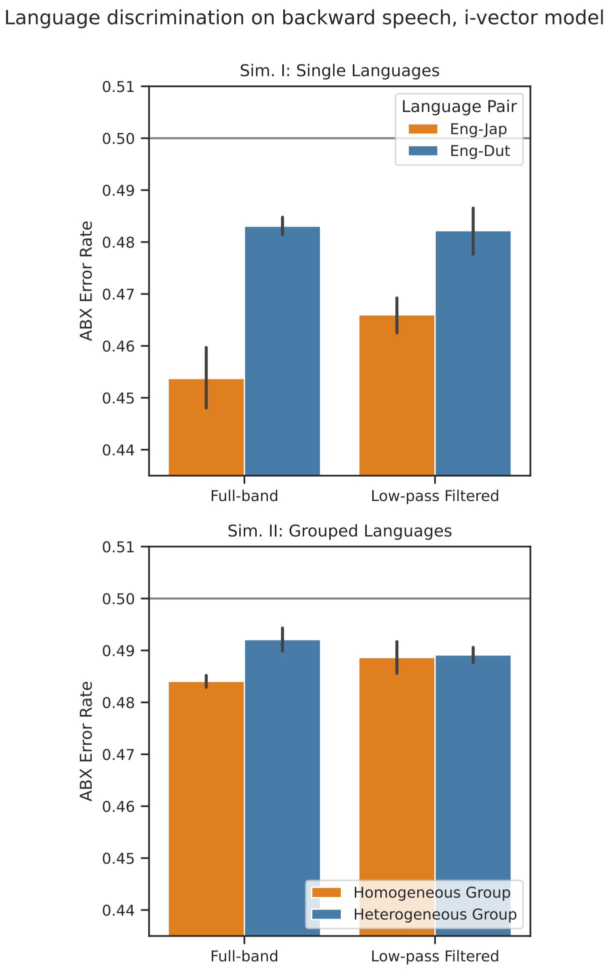

Figure 2: Backward speech results on the i-vector models. A lower ABX error rate indicates better discrimination, where chance level (50%) is marked in grey. Errorbars represent 95% CI calculated over models trained on disjoint training data.

discrimination. For the i-vector model, the backward speech results are almost identical to the results on forward speech.
